# Inequities in Medical Debt and Its Contributing Health Care Services in New York City

**DOI:** 10.1001/jamanetworkopen.2026.0608

**Published:** 2026-03-04

**Authors:** Judy Fordjuoh, Rachel Schwartz, Duncan Maru, Rishi Sood, Jenai Jackson, Ellen Weiss Wiewel

**Affiliations:** 1 Bureau of Equitable Health Systems, The Center for Health Equity and Community Wellness, New York City Department of Health, New York, New York

## Abstract

This cross-sectional study estimates the prevalence of and factors associated with medical debt among adults in New York, New York, using citywide survey data.

## Introduction

Medical debt remains a barrier to health and financial well-being in the US, affecting over 100 million people.^1^ Debt disproportionately impacts low-income individuals, the uninsured, and traditionally marginalized communities.^2,3^ Despite expanded coverage under the Patient Protection and Affordable Care Act, 6% of New York, New York (NYC), residents remain uninsured.^4^ High deductibles and copayments can result in unpaid bills even among insured individuals.^5^

In New York State, approximately 740 000 adults had medical debt in collections as of 2022,^6^ although this likely underestimates the true burden. We estimated the prevalence of and factors associated with medical debt among NYC adults using citywide survey data.

## Methods

This cross-sectional study and the Health Opinion Poll survey were approved by the NYC Department of Health and Mental Hygiene (DOHMH) institutional review board. Participants provided informed consent. We followed the STROBE reporting guidelines.

We analyzed data from the April 2023 DOHMH Health Opinion Poll, a weighted survey representative of NYC adults. Respondents reported whether they or a family member had over $500 in unpaid medical bills and identified the medical services contributing to those bills. Analyses were weighted to match NYC’s adult population by borough, sex, age, race and ethnicity, and education.

Race and ethnicity were self-reported, and race categories were defined by DOHMH. Latino/a included respondents identifying as Latino/a regardless of race, and the Other category included multiracial and Indigenous respondents.

We estimated the prevalence of medical debt and, among those with debt, services contributing to debt by sociodemographic characteristics. Data cleaning was conducted in SAS statistical software version 8.3 (SAS Institute), and logistic regression models were performed using SUDAAN statistical software version 11.0.4 (RTI International). Model 1 assessed the presence of medical debt. Models 2, 3, and 4 assessed debt attributed to hospitalization and emergency or urgent care (model 2); dental care (model 3); and physician visits or nonemergency procedures, surgical procedures, tests, or treatments (model 4). Each model compared debt from the specified service with no debt from that service or no medical debt at all.

Variables were included on the basis of prior literature and model convergence. Risk ratios and 95% CIs were estimated using logistic regression between each covariate and outcome. Adjusted models controlled for age, gender, race and ethnicity, education, insurance status, and household poverty. Statistical significance was defined as a 95% CI that excluded 1.

## Results

Among 1250 respondents (weighted number, 5.8 million), 13.2% (weighted number, 769 000) reported medical debt exceeding $500 (Table 1). The survey response rate was 42%. Respondents were predominantly aged 25 to 44 years, cisgender women, White, college educated, and insured. Medical debt prevalence was highest among adults aged 18 to 24 years (34.2%) and uninsured individuals (31.2%) and was more common among Latino (19.1%) and adults in the other race group (23.2%) than White adults (9.1%).

**Table 1.** Prevalence of Medical Debt by Selected Characteristics^a^

Characteristic	No. of respondents			With medical debt, % (95% CI)	RR (95% CI)	
	Unweighted	Weighted^b^	Weighted with medical debt^b^		Unadjusted	Adjusted^c^
Overall	1250	5 817 000	769 000	13.2 (10.1-17.0)	NA	NA
Age group, y						
18-24	23	329 000	112 000	34.2 (14.5-61.4)^d^	3.35 (1.40-8.06)	2.52 (0.84-7.50)
25-44	488	2 415 000	246 000	10.2 (6.3-16.2)	1 [Reference]	1 [Reference]
45-64	446	1 750 000	321 000	18.4 (12.9-25.4)	1.80 (1.01-3.23)	1.72 (1.05-2.83)^e^
≥65	293	1 323 000	89 000	6.7 (3.2-13.8)^d^	0.66 (0.27-1.60)	0.82 (0.31-2.19)
Gender						
Cisgender man	439	2 758 000	386 000	14.0 (9.1-20.8)	1 [Reference]	1 [Reference]
Cisgender woman	797	2 989 000	352 000	11.8 (8.5-16.1)	0.84 (0.50-1.42)	0.93 (0.59-1.47)
Transgender, nonbinary, or another gender identity not listed	14	70 000	31 000	44.5 (13.4-80.6)^d^	3.18 (1.17-8.65)	3.52 (1.78-6.96)^e^
Race and ethnicity						
Asian or Pacific Islander	203	916 000	47 000	5.1 (2.1-12.1)^d^	0.56 (0.20-1.62)	0.55 (0.23-1.33)
Black, non-Latino	198	1 168 000	198 000	17.0 (10.3-26.6)	1.86 (0.88-3.92)	1.56 (0.91-2.67)
Latino	270	1 529 000	292 000	19.1 (12.1-28.8)	2.09 (1.02-4.31)	1.26 (0.67-2.38)
White, non-Latino	534	1 991 000	182 000	9.1 (5.1-15.9)	1 [Reference]	1 [Reference]
Other^f^	45	213 000	49 000	23.2 (10.3-44.3)^d^	2.54 (0.99-6.51)	2.20 (0.97-4.98)
Education^g^						
High school diploma or less	215	2 252 000	426 000	18.9 (12.3-28.0)	2.27 (1.39-3.70)	1.56 (0.92-2.64)
Some college	235	1 287 000	153 000	11.9 (7.4-18.4)	1.43 (0.84-2.41)	1.09 (0.64-1.88)
Bachelor’s degree and higher	800	2 279 000	190 000	8.3 (6.4-10.8)	1 [Reference]	1 [Reference]
Insurance type^h^						
Public insurance	442	2 253 000	259 000	11.5 (7.3-17.7)	1 [Reference]	1 [Reference]
Private insurance	630	2 326 000	220 000	9.4 (6.6-13.3)	0.82 (0.47-1.44)	1.15 (0.47-2.79)
Insured but insurance type not identified	105	673 000	95 000	14.1 (6.8-27.1)^d^	1.23 (0.54-2.81)	1.11 (0.46-2.67)
Do not know their insurance status	NA^i^	NA^i^	NA^i^	NA^i^	4.14 (1.22-14.01)	4.10 (0.83-20.17)
Uninsured	65	456 000	142 000	31.2 (15.6-52.5)^d^	2.71 (1.27-5.76)	2.77 (1.36-5.61)^e^
Household poverty						
Low income (<200% FPL)	388	2 513 000	424 000	16.9 (11.6-23.8)	1.62 (0.96-2.72)	1.13 (0.57-2.22)
Medium or high income (≥200% FPL)	862	3 305 000	345 000	10.4 (7.1-15.0)	1 [Reference]	1 [Reference]

Abbreviations: FPL, federal poverty level; NA, not applicable; RR, risk ratio.

^a^ Data are from the NYC Health Opinion Poll (HOP) 2023, collected from April 10 to 26, 2023.

^b^ Population estimates have been rounded to the nearest thousand.

^c^ Adjusted RRs have been adjusted to control for age, gender, race and ethnicity, education, insurance type, and household poverty.

^d^ Estimate should be interpreted with caution. Estimate’s relative SE (a measure of estimate precision) is greater than 30%, or the 95% CI half-width is greater than 10 or the sample size is too small, making the estimate potentially unreliable.

^e^ Denotes a 95% CI that does not cross the null value.

^f^ Other includes multiracial and Indigenous respondents.

^g^ The education variable has been recoded to reduce the number of levels.

^h^ The insurance type variable was recoded using insurance data from Health NYC registration and responses to insurance status questions fielded in HOP 20.

^i^ Data are suppressed owing to imprecise and unreliable estimates.

In adjusted models (Table 1), adults aged 45 to 64 years were almost twice as likely to have debt than those aged 25 to 44 years. Persons identifying as transgender, nonbinary, or another gender identity (TGNB) were 3 times as likely to have debt compared with cisgender men. Uninsured adults were almost 3 times as likely to have medical debt as those with public insurance.

Among respondents with debt, the most frequently cited sources were physician visits, nonemergency procedures, or treatments; hospitalization or emergency or urgent care; and dental care (Table 2). After adjustment, debt from these services was more likely among younger adults, TGNB individuals, Black and Latino adults, and uninsured persons. Debt from hospitalizations or emergency and urgent care was highest among respondents in the other race group, uninsured individuals, and TGNB adults, whereas dental debt was less common among adults aged 65 years and older.

**Table 2.** Prevalence of Service Types Contributing to Medical Debt by Selected Characteristics^a^

Characteristic	Model 2^b^			Model 3^b^			Model 4^b^		
	Percentage^c^	Unadjusted RR (95% CI)	Adjusted RR (95% CI)^d^	Percentage^c^	Unadjusted RR (95% CI)	Adjusted RR (95% CI)^d^	Percentage^c^	Unadjusted RR (95% CI)	Adjusted RR (95% CI)^d^
Age group, y									
18-24	37.6	2.09 (0.53-8.29)	1.26 (0.28-5.74)	44.3	2.62 (0.65-10.58)	1.65 (0.27-10.10)	79.7	5.39 (1.84-15.76)	4.12 (1.28-13.25)^e^
25-44	60.2	1 [Reference]	1 [Reference]	56.6	1 [Reference]	1 [Reference]	49.6	1 [Reference]	1 [Reference]
45-64	39.4	1.17 (0.48-2.84)	1.27 (0.71-2.29)	43.5	1.38 (0.50-3.79)	1.56 (0.69-3.52)	57.0	2.06 (0.97-4.35)	1.67 (0.86-3.23)
≥65	31.2	0.31 (0.08-1.16)	0.43 (0.12-1.49)	9.3	0.10 (0.03-0.37)	0.14 (0.03-0.55)^e^	75.7	1.01 (0.34-3.00)	1.38 (0.46-4.19)
Gender									
Cisgender man	34.5	1 [Reference]	1 [Reference]	53.9	1 [Reference]	1 [Reference]	52.0	1 [Reference]	1 [Reference]
Cisgender woman	51.4	1.28 (0.51-3.21)	1.87 (0.89-3.94)	35.5	0.56 (0.25-1.29)	0.78 (0.37-1.64)	65.5	1.05 (0.49-2.25)	0.99 (0.53-1.83)
Transgender, nonbinary, or another gender identity not listed	100.0	9.46 (2.78-32.23)	11.15 (4.32-28.73)^e^	26.8	1.62 (0.22-11.98)	1.42 (0.09-23.30)	100.0	6.12 (2.05-18.31)	6.23 (2.92-13.26)^e^
Race and ethnicity									
Asian or Pacific Islander	70.1	0.78 (0.16-3.91)	0.77 (0.25-2.40)	67.1	0.75 (0.15-3.83)	0.77 (0.25-2.42)	66.8	1.14 (0.29-4.46)	0.99 (0.30-3.27)
Black, non-Latino	36.4	1.28 (0.38-4.24)	1.24 (0.53-2.93)	26.3	0.92 (0.27-3.22)	0.94 (0.37-2.41)	67.7	3.80 (1.67-8.65)	2.35 (1.13-4.87)^e^
Latino	34.4	1.43 (0.40-5.06)	0.74 (0.33-1.64)	47.9	2.00 (0.55-7.26)	1.12 (0.44-2.86)	70.0	4.43 (2.03-9.68)	2.23 (1.01-4.89)^e^
White, non-Latino	51.1	1 [Reference]	1 [Reference]	50.9	1 [Reference]	1 [Reference]	33.5	1 [Reference]	1 [Reference]
Other^f^	95.1	4.79 (1.29-17.76)	4.55 (1.95-10.62)^e^	46	2.33 (0.48-11.31)	2.38 (0.82-6.91)	61.9	4.75 (1.57-14.43)	2.89 (0.79-10.50)
Education^g^									
High school diploma or less	46.1	2.18 (1.01-4.67)	1.78 (0.86-3.69)	46.9	2.78 (1.24-6.24)	1.74 (0.81-3.77)	58.6	2.60 (1.30-5.18)	1.52 (0.79-2.91)
Some college	37.2	1.03 (0.48-2.21)	1.18 (0.54-2.57)	43.6	1.52 (0.56-4.14)	1.40 (0.61-3.23)	74.6	2.08 (1.04-4.14)	1.25 (0.65-2.37)
Bachelor’s degree and higher	48.7	1 [Reference]	1 [Reference]	38.8	1 [Reference]	1 [Reference]	51.8	1 [Reference]	1 [Reference]
Insurance type^h^									
Public	31.2	1 [Reference]	1 [Reference]	36.7	1 [Reference]	1 [Reference]	62.7	1 [Reference]	1 [Reference]
Private	41.4	1.12 (0.51-2.44)	1.31 (0.52-3.30)	31.6	0.73 (0.31-1.72)	0.69 (0.16-3.06)	66.4	0.86 (0.39-1.92)	1.76 (0.63-4.89)
Insured but insurance type not identified	75.0	3.07 (1.02-9.17)	2.60 (0.96-7.06)	57.1	1.98 (0.60-6.55)	1.82 (0.44-7.47)	49.7	0.98 (0.25-3.78)	0.71 (0.17-3.02)
Do not know their insurance status	100.0	13.75 (3.75-50.46)	15.80 (5.83-42.85)^e^	100.0	11.70 (3.06-44.79)	8.06 (14.5-44.68)^e^	0.0	6.93 (3.08-15.58)	8.51 (2.41-30.06)^e^
Uninsured	34.4	3.09 (0.99-9.62)	3.84 (1.44-10.28)^e^	48	3.68 (0.94-14.37)	2.57 (0.63-10.47)	74.9	3.24 (1.17-8.93)	3.69 (1.61-8.48)^e^
Household poverty									
Low income (<200% FPL)	39.9	1.30 (0.58-2.92)	1.11 (0.52-2.36)	43.4	1.60 (0.63-4.09)	0.95 (0.28-3.22)	64.8	1.94 (0.98-3.84)	1.29 (0.56-2.96)
Medium or high income (≥200% FPL)	51.5	1 [Reference]	1 [Reference]	45.4	1 [Reference]	1 [Reference]	54.4	1 [Reference]	1 [Reference]

Abbreviations: FPL, federal poverty level; RR, risk ratio.

^a^ Data are from the NYC Health Opinion Poll (HOP) 2023, collected from April 10 to 26, 2023.

^b^ Model 2 refers to hospitalization, emergency care, or urgent care (prevalence, 45.0%; 95% CI, 31.5%-59.4%). Model 3 refers to dental care (prevalence, 44.0%; 95% CI, 30.5%-59.1%). Model 4 refers to physician visits and nonemergency medical procedures, surgical procedures, tests, or treatments (prevalence, 60.0%; 95% CI, 45.9%-72.8%).

^c^ Among those with health care debt, the values indicate the percentage whose debt was owing to this medical service.

^d^ Adjusted RRs are estimated among individuals with reported debt and examine the association between having a specific type of service contributing to debt and respondent characteristics, adjusting for age, gender, race and ethnicity, education, insurance type, and household poverty.

^e^ Denotes a 95% CI that does not cross the null value.

^f^ Other includes multiracial and Indigenous respondents.

^g^ The education variable has been recoded to reduce the number of levels.

^h^ The insurance type variable was recoded using insurance data from Health NYC registration and responses to insurance status questions fielded in HOP 20.

## Discussion

This cross-sectional study found that approximately three-quarters of a million adults in NYC have medical debt exceeding $500, indicating substantial financial strain that may delay or deter care. Although service frequency and cost were not measured, our findings suggest that medical debt extends beyond emergency or hospital care. Policies expanding affordable coverage, improving billing transparency, and recent debt cancellation and collection reforms may reduce these burdens.

Limitations include that data were self-reported and may be subject to recall or social desirability bias. Also, insurance information may have changed between registration and survey completion, introducing misclassification. Excluding respondents with missing demographic data and a small sample size may have underestimated medical debt and limited precision.

## Notes

Supplement. Data Sharing Statement
