# Supplementary material for: Inequities in Medical Debt and Its Contributing Health Care Services in New York City
Source: JAMA Netw Open. 2026 Mar 4;9(3):e260608. doi: 10.1001/jamanetworkopen.2026.0608 (PMC12961514; doi:10.1001/jamanetworkopen.2026.0608)
Supplement: Supplement. — Data Sharing Statement [file jamanetwopen-e260608-s001.pdf]

Data Sharing Statement

Fordjuoh. Inequities in Medical Debt and Its Contributing Health Care Services in New York City. *JAMA Netw Open*. Published March 04, 2026. doi:10.1001/jamanetworkopen.2026.0608

Data
Data available: No

Additional Information
Explanation for why data not available: Due to the nature of the research, supporting data is not available.
